# 2017 ISCB Innovator Award: Aviv Regev

**DOI:** 10.12688/f1000research.11585.1

**Published:** 2017-06-26

**Authors:** Christiana N. Fogg, Diane Kovats, Bonnie Berger

**Affiliations:** 1 Freelance Writer, Kensington, MD, USA; 2 International Society for Computational Biology, Bethesda, MD, 20814, USA; 3 Department of Mathematics, Massachusetts Institute of Technology, Cambridge, MA, 02139, USA

**Keywords:** Editorial

## Abstract

2017 marks the second year of the International Society for Computational Biology (ISCB) Innovator Award, which recognizes an ISCB scientist who is within two decades of having completed his or her graduate degree and has consistently made outstanding contributions to the field. The 2017 winner is Dr. Aviv Regev, Professor of Biology at the Massachusetts Institute of Technology (MIT), a Core Member and Chair of the Faculty of the Broad Institute of MIT and Harvard, and an HHMI Investigator. Regev will receive her award and deliver a keynote address during International Conference on Intelligent Systems for Molecular Biology/European Conference on Computational Biology (ISMB/ECCB) 2017 in Prague, Czech Republic (July 21 - 25, 2017).

## Aviv Regev: Seeing cells as life’s smallest circuits

Aviv Regev first pursued her studies in a unique interdisciplinary program at Tel Aviv University, where she planned to focus on maths and computer science ^1^. But she discovered her interest in biology in the classroom of evolutionary biologist Eva Jablonka. Regev said, “I found biology because of her – in my first year as an undergrad, I took a genetics course with her in what is now called the ‘flipped classroom’ style. It was all abstract and inferential, and I was hooked.”

Before starting her PhD thesis at Tel Aviv University, Regev began to really think about cells as computers, particularly how they are comprised of circuits. Regev’s deep interest in this concept started at a conference where new approaches for modeling concurrent computation were featured, and she immediately considered this as a way to model cell circuitry. She was able to develop her ideas into a PhD project under the mentorship of Udi Shapiro and Eva Jablonka, and she recalled, “No one was working on this type of project. I did, however, have the great fortune to find Udi, who listened to my idea. He thought it was important. He didn’t want to work on it himself – but he wanted *me* to be able to work on it.”

Regev completed her PhD in 2002 and was selected to be a Bauer Fellow at the Center for Genomics Research at Harvard University, which gave her an intellectual community, as well as freedom and funding to build a small independent research group. She continued to pursue her interest in modeling cell circuits using gene expression and genomic data, and she developed with her colleagues several widely used algorithms and computational tools, including Module Networks and Synergy. She received early support from Andrew Murray at Harvard University, who shared Regev’s view that it was critical to deeply understand both theory and experiments.

In 2006, Regev was given a joint faculty appointment at MIT and the Broad Institute, and she started applying her cell circuit modeling algorithms to understanding different cell types, particularly cells of the immune system. Once again, Regev struck out on an independent line of research. She recalled, “Many people were not focused on circuits. But that was OK. I wanted to build and be part of a community that would open a new direction.” Eric Lander at Broad – a longtime supporter of female and young scientists with leadership potential -- stood behind and supported Regev’s independent scientific vision at this critical point in her career.

Regev’s independent research program has blossomed since she founded her lab, and she has applied her interest in how cellular circuits function and rewire to a wide range of biological questions, including how immune cells rapidly respond and differentiate, how hematopoietic stem cells develop into different blood cells, and how evolutionary changes occur over millions of years. She is both a computational biologist with keen instincts about how to extract insight from data, and an experimental biologist with the ability to create new methods and deploy cutting edge technology to address fundamental questions.

**Figure:**
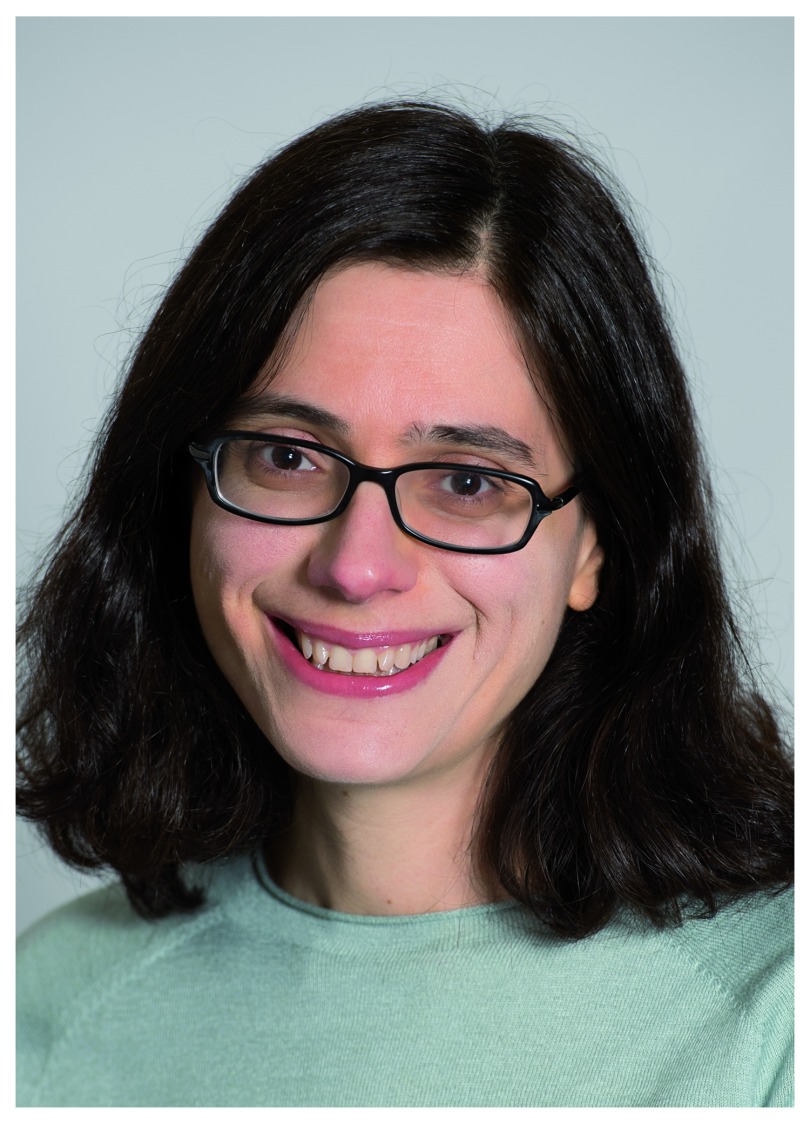
Aviv Regev

Regev continues to be drawn to seemingly intractable problems, such as biological scenarios with a massive number of hypothetical combinations or interactions, and making them into manageable problems by using sampling approaches. Her work on cells of the immune system reflects this focus, and she recalls one of her most unexpected findings emerged in 2012 while working with collaborators on applying single-cell RNA-seq to the analysis of dendritic cells. In contrast to present day technology, which enables the profiling of thousands of cells quickly and cheaply, this study only looked at 18 cells and required a tremendous effort. Regev recalled, “What we found was surprising in two ways. First, we were examining just one cell type which we thought was well-defined, so we did not expect to find major differences in gene expression between the cells – yet we saw 1,000-fold differences, from which we could recover regulatory molecules that accounted for this variation. Second, we discovered surprising patterns in alternative splicing – some cells preferentially used one isoform, others used another. We had been expecting the cells to use both. This added up to a bigger surprise: we weren’t really looking at one group of cells. We were looking at two subgroups, which we now know represent different developmental programs. A great deal of my work now focuses on understanding heterogeneity of this type – defining and understanding cells at a much higher resolution than we could before.”

Regev has passed along her love of science through her mentorship of postdocs, graduate students, and undergraduates, and outside of the lab she has maintained an intense teaching load and worked to overhaul the undergraduate genetics course to include quantitative content. She is grateful to her mentors who gave her freedom to pursue her own scientific interests and this has guided her style of mentorship. She said, “Today, when I see a person with an idea, I don’t care about career stage -- maybe they’re a grad student or an undergrad; maybe they are a seasoned staff scientist. I care about who they are. Do they show the seeds of independence, vision, and leadership? And what is their idea? If it’s challenging in entirely new ways, and can transform the world, it should be grown. As I mentor my students and postdocs, I try to let them spread their own wings – to be their colleague and collaborator.”

At Broad, Regev was recently appointed Chair of the Faculty, and in this role she has been focusing on initiatives to strengthen and build communities around computational biology and advance software engineering approaches to biological data analysis. She has served the greater computational biology community in many ways through work on numerous advisory boards, journal editorial boards, and program committees for conferences. Regev has been a reviewing editor for eLife since its inception, and more recently a senior editor with a major responsibility for computational biology, genomics and theory papers.

Regev is gratified by her selection for the 2017 ISCB Innovator Award, and she said, “Biology is such a data science now, and ISCB is the community that made that happen – so it is especially exciting and gratifying to be receiving such an honor from peers in this community.”

## Footnote

^1^ https://www.hhmi.org/scientists/aviv-regev

